# Tips and Tricks of Orbital Atherectomy in Real-World Clinical Practice

**DOI:** 10.3390/jcm15134913

**Published:** 2026-06-24

**Authors:** Takashi Ashikaga, Toshihiro Nozato, Yasutoshi Nagata, Tetsumin Lee, Masakazu Kaneko, Toru Misawa, Masashi Nagase, Mao Matsuyama, Daigo Kachi, Maki Ohira, Kazuki Matsuda

**Affiliations:** Department of Cardiology, Japanese Red Cross Musashino Hospital, 1-26-1 Kyonan-cho, Musashino City 180-8610, Tokyo, Japan

**Keywords:** orbital atherectomy, percutaneous coronary intervention, coronary artery calcification, IVUS, OCT

## Abstract

Orbital atherectomy (OA) is a highly effective atherectomy device used to treat heavily calcified coronary arteries. The technique for using OA is critical and depends on appropriate use of the dedicated guidewire. OA employs a centrifugal, differential sanding mechanism with bidirectional movement. When used with proper technique, the device appears to be associated with a low rate of complications, such as bradycardia and slow flow, compared with rotational atherectomy (RA), and results in high procedural success rates. We describe our experience with the OA device and procedural techniques in our catheterization laboratory.

## 1. Introduction

Percutaneous coronary intervention (PCI) for severely calcified lesions has been shown to be associated with a higher incidence of complications and poorer long-term prognosis compared with noncalcified lesions. In general, moderate-to-severe calcification accounts for approximately 30% of PCI cases, whereas severe calcification accounts for 6–20% [1,2]. In the presence of significant calcification, PCI may fail because of balloon crossing failure, inadequate balloon expansion, balloon rupture, or stent delivery failure. Even when drug-eluting stent (DES) implantation is successful, severely calcified lesions have been reported to be associated with a significantly poorer prognosis compared with nonseverely calcified lesions. Possible mechanisms include incomplete stent expansion, asymmetric stent expansion, polymer damage, and stent deformation during deployment. Stent underexpansion, stent malapposition, and stent edge dissection are also known to increase the risk of in-stent restenosis and stent thrombosis. There are several different calcium modification strategies available, whether intravascular lithotripsy (IVL) or atherectomy devices, such as rotational atherectomy (RA), orbital atherectomy (OA), excimer laser atherectomy (ELCA). These atherectomy devices are established for the modification of coronary calcification [3,4]. Here, we describe the tips and tricks of OA in clinical practice.

## 2. Orbital Atherectomy and Clinical Studies

Rotational atherectomy (RA; Boston scientific, Natick, MA, USA) and orbital atherectomy (OA: Abbott Vascular, St. Paul, MN, USA) have been used as plaque-modifying devices for calcified lesions. RA was introduced more than 40 years ago, and numerous reviews and consensus statements have been published. Optimization and standardization of RA techniques have been facilitated to achieve high-quality RA [5]. In contrast, OA was introduced 15 years ago and only some clinical studies have been reported in the literature [6,7,8,9,10]. The ORBIT I Trial indicated that OA may serve as an effective strategy for modifying lesion compliance in severely calcified lesions. The ORBIT II Trial indicated the significant increase in mean minimal lumen diameter on quantitative coronary angiography. ECLIPSE Trial was a multicenter, open-label, randomized control trail comparing the OA with balloon angioplasty before drug-eluting stent implantation in patients with severely calcified coronary lesions. The findings of this study indicate target vessel failure at 1 year in 11.5% of patients in the OA group compared to 10.0% in the balloon angioplasty group, with no statistically significant difference. The ECLIPSE Trial also demonstrated the importance of image-guided PCI as superior to angio-guided PCI. Since the mechanism of OA is different from that of RA, OA tips and tricks remain less known.

## 3. OA Action Mechanisms 

In RA, a burr coated with diamond particles is attached to the distal end of the drive shaft, which is rotated by compressed nitrogen delivered to the turbine at the proximal end. RA enlarges the lumen through mechanical modification of plaque along the guidewire bias and by reducing plaque rigidity, thereby facilitating subsequent dilation. Although various RA techniques have been introduced into clinical practice, the modification effect depends on the burr size [11,12]. Operators should be aware of the risk of the slow-flow phenomenon, which may result from increased platelet aggregation and distal embolization [13]. Two types of guidewires are available—extra-support and floppy—and the appropriate type should be selected according to lesion characteristics.

In contrast, OA utilizes a 1.25 mm eccentrically mounted, diamond-coated crown that rotates in an expanding lateral orbit as centrifugal force increases, resulting in differential sanding of coronary calcification. OA is primarily powered by electricity and is designed to maintain a constant rotational speed. Only one guidewire, named Viperwire, is available. Unlike RA, it is difficult to use a reduction in rotational speed as an indicator of plaque resistance; therefore, changes in sound and tactile feedback perceived at the advancer knob are important during OA treatment. The OA crown enables both antegrade and retrograde modification and maintains continuous blood flow. OA provides an effective sanding mechanism and has the advantage of preserving luminal patency during treatment, thereby reducing hemodynamic instability, minimizing the risk of thermal injury to the vessel, and producing minuscule particulates associated with a lower risk of slow- or no-reflow [14,15]. Furthermore, the incidence of bradycardia related to ischemia is low, and in most cases, a temporary pacemaker is not required [16].

**Figure 1:**
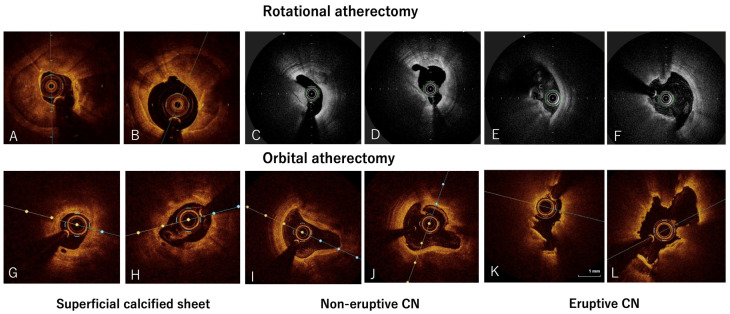
Modification effects of rotational atherectomy and orbital atherectomy. Modification of rotational atherectomy is guidewire-biased. Modification of orbital atherectomy is both guidewire-biased and non-guidewire-biased by centrifugal force. (**A**,**B**) Superficial calcified sheet treated with 2.0 mm rotational atherectomy. (**C**,**D**) Noneruptive calcified nodule treated with 1.75 mm rotational atherectomy. (**E**,**F**) Eruptive calcified nodule treated with 1.75 mm rotational atherectomy. (**G**,**H**) Superficial calcified sheet treated with orbital atherectomy. (**I**,**J**) Noneruptive calcified nodule treated with orbital atherectomy. (**K**,**L**) Eruptive calcified nodule treated with orbital atherectomy.

**Figure 2:**
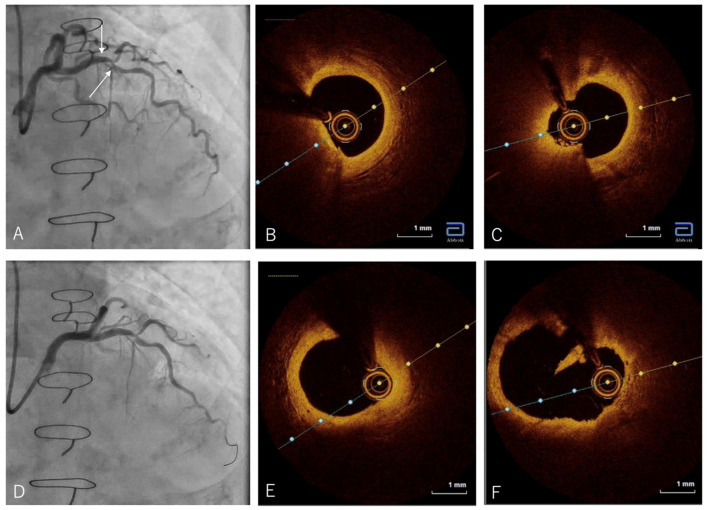
Complication of OA (vessel injury). (**A**) Angiogram before OA. (**B**,**C**) The guidewire was positioned near eccentric calcification, which was effectively modified by OA at 80,000 rpm. (**D**) Angiogram after OA. (**E**,**F**) The guidewire was positioned near fibrous plaque. Vessel injury occurred after OA at 80,000 rpm. White arrows indicate the lesion.

Both RA and OA can modify calcified lesions along the guidewire bias because these devices are advanced into the coronary vessel over a guidewire. In addition, OA can modify plaque both along and beyond the guidewire bias because the OA crown can move with centrifugal force (Figure 1). Even in cases in which the guidewire is positioned away from the area of maximal plaque burden, such as in eruptive or non-eruptive calcified nodules, larger plaque modification may be achieved with OA than with RA. In eccentrically calcified lesions with the guidewire adjacent to the calcified segment, modification of calcification improves subsequent balloon dilatation and facilitates successful DES implantation. Conversely, when normal vessel segments or noncalcified plaque are located near the guidewire, treatment with OA may be hazardous because centrifugal force may result in greater modification of noncalcified tissue compared with RA (Figure 2) [17]. Therefore, it is important to confirm the location and extent of ablation using intravascular imaging to reduce the risk of complications such as pseudoaneurysm and perforation (Table 1).

**Figure 3:**
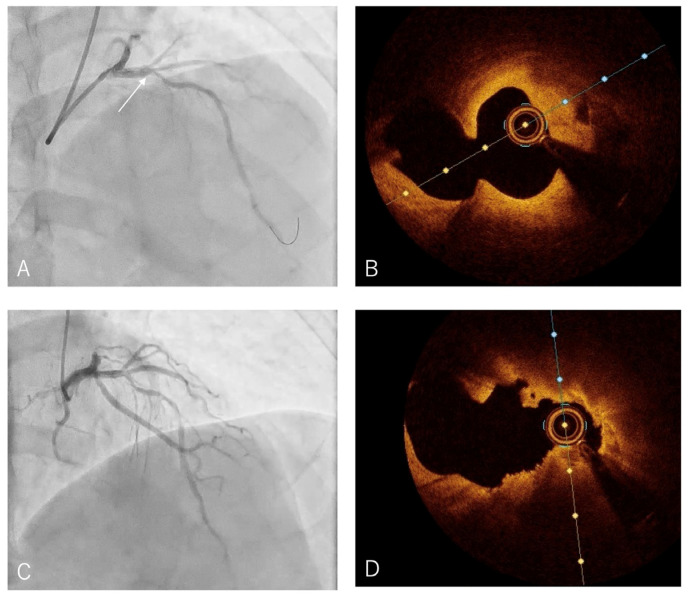
OA for deep calcium. (**A**) Angiogram. (**B**) Optical coherence tomography (OCT) demonstrating deep calcium at a bifurcation lesion. (**C**) Angiogram after OA at 120,000 rpm. (**D**) Deep calcium successfully modified after OA at 120,000 rpm. The white arrow indicates the lesion.

In general, deep calcium is not considered an ideal indication for plaque-modifying devices. IVL can permit circumferential fracture of deep calcium through acoustic shockwave and improve vessel compliance [18]. Kini et al. demonstrated that deeper tissue modification and better stent apposition could be achieved with OA compared with RA [19]. OA can modify deep calcium via centrifugal force and achieve greater lumen gain if the guidewire is located near deep calcium (Figure 3).

### 3.1. Technical Issues of OA

Whereas the RA burr is ideally advanced using a slow pecking motion, the OA crown is best advanced with a slow, continuous movement, with the option to decelerate in segments requiring greater ablation—an approach referred to as the engage-and-release technique. A regular coronary guidewire is first advanced distal to the lesion. If an intravascular imaging device can be passed, the target debulking area is determined based on imaging findings. The guidewire is then typically exchanged for a ViperWire using a microcatheter. Because the ViperWire is relatively easy to manipulate, it can often be advanced to the distal vessel even using the bare-wire technique when a microcatheter cannot be passed. The Glide Assist mode (5000 rpm), which provides an excellent crossing profile, may facilitate lesion crossing when insertion of an imaging device is not feasible. It is important to position the OA catheter platform, as the guidewire may be adjacent to normal tissue distal to the target segment, thereby increasing the risk of vessel injury. During antegrade OA, the catheter should be advanced using the engage-and-release technique, with slow, controlled back-and-forth movement from a stable platform positioned slightly proximal to the culprit lesion, similar to the technique used for RA. The OA crown should be advanced slowly, typically at a speed of 1 mm/s or less. The maximum run time is approximately 30 s, and an audible warning is emitted 25 s after activation, providing procedural feedback. After OA at 80,000 rpm, the calcified segment should be reassessed using intravascular imaging to determine whether additional modification at 120,000 rpm is required.

### 3.2. Guide Catheter Selection and Precautions When Using a Guide Extension Catheter

The OA crown is available in a single size, and a 6 F guide catheter is generally sufficient to accommodate most lesions. Use of a 6 F or larger guide catheter allows contrast injection during OA modification to confirm the position of the OA catheter and distal coronary flow. Based on its low crossing profile, a 5 F guide catheter may also be used in selected cases.

**Figure 4:**
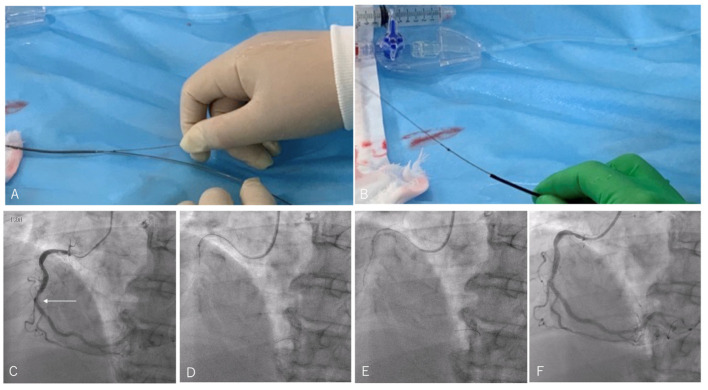
Technique using a 6 F guide extension catheter. (**A**,**B**) The orbital crown was advanced within the 6 F guide extension catheter outside the body and then introduced into the coronary system. (**C**) Angiogram demonstrating a tortuous right coronary artery lesion. (**D**) Both the orbital crown and the guide extension catheter were advanced within the right coronary artery. (**E**) Glide Assist mode enabled the crown to cross the tortuous lesion. (**F**) Angiogram after OA at 80,000 rpm. The white arrow indicates the culprit lesion.

When a 6 F guide extension catheter is used, it is often necessary to advance the OA catheter into the extension catheter in an assembled configuration outside the patient. In this situation, the OA crown extends beyond the tip of the guide extension catheter, permitting advancement in Glide Assist mode (Figure 4).

### 3.3. Treatment Strategies for OA

#### 3.3.1. IVUS Guidance

**Figure 5:**
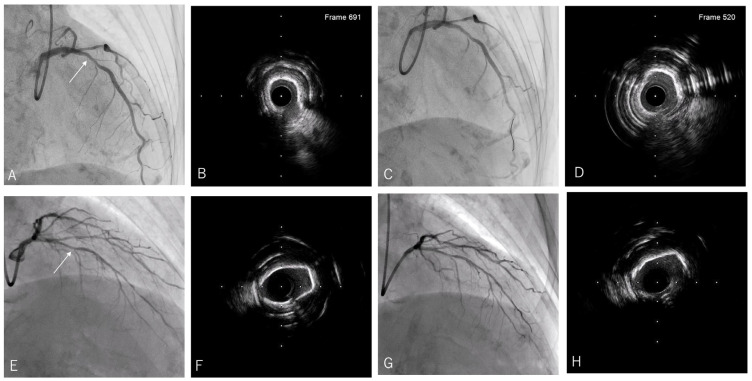
IVUS-guided OA. (**A**) Angiogram. (**B**) IVUS demonstrating severe calcification. (**C**) Angiogram after OA at 120,000 rpm. (**D**) IVUS demonstrating reverberations and lumen gain. (**E**) Angiogram. (**F**) IVUS demonstrating severe calcification. (**G**) Angiogram after OA at 120,000 rpm. (**H**) IVUS demonstrating disappearance of calcium without reverberation. White arrows indicate the lesion.

**Figure 6:**
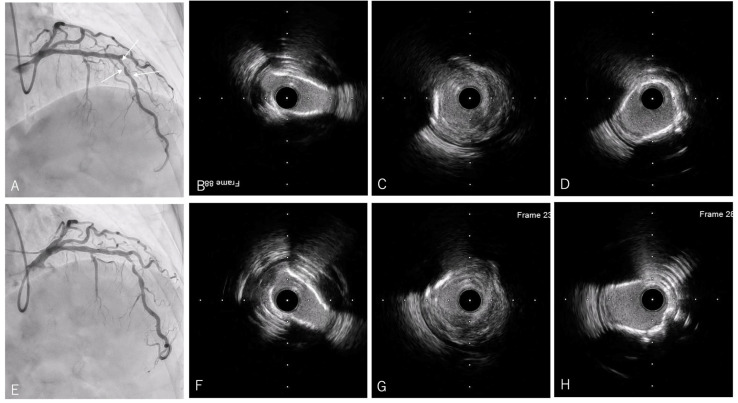
OA with IVUS guidance in a severely calcified LAD lesion. (**A**) Angiogram. (**B**–**D**) Intravascular ultrasound (IVUS) demonstrating severe calcification in the proximal and distal segments. However, the guidewire position was not good for OA (10 o’clock) in the midportion. The IVUS marking method identified the OA sites in the proximal and distal segments and no touch in the midportion. (**E**–**H**) Angiogram after OA at 80,000 rpm. Lumen gain could be obtainedand reverberations occurred from 12 to 2 o’clock and 6 to 8 o’clock in the proximal lesion. Lumen gain could be obtained, and reverberations occurred from 12 to 2 o’clock in the distal lesion. In the midportion, OA crown was passed with Glideassist mode. No significant change was observed in the midportion. White arrows indicate the calcified and non-calcified lesions.

**Figure 7:**
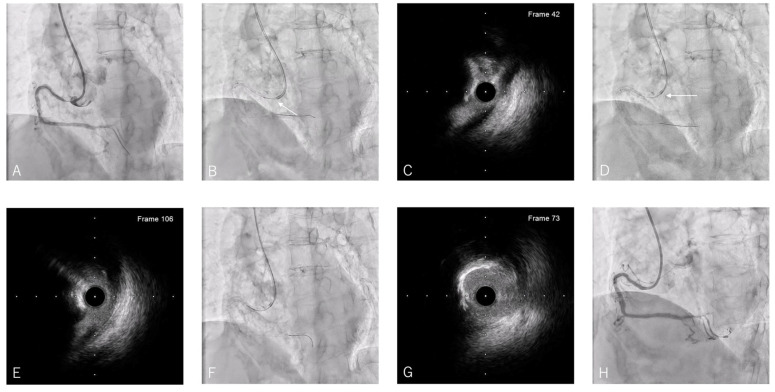
OA in an ostial RCA lesion. (**A**) Angiogram demonstrating ostial right coronary artery (RCA) narrowing. (**B**,**C**) IVUS demonstrating that the guidewire was positioned away from the eccentric calcified lesion in this guide catheter configuration. (**D**,**E**) IVUS demonstrating that the guidewire was positioned near the eccentric calcified lesion after adjustment of guide catheter position. (**F**–**H**) OA at 80,000 rpm was performed in this guide catheter position, and subsequent IVUS and angiography demonstrated effective plaque modification. White arrows indicate the position of the guide catheter.

If the guidewire is positioned adjacent to the calcified lesion, OA can generally be performed safely. Because the thickness of calcification cannot be precisely measured by intravascular ultrasound (IVUS), the effectiveness of OA is primarily assessed based on reverberation artifacts and lumen enlargement. However, in some cases, the calcified plaque itself may become less apparent; therefore, reliance solely on reverberation should be avoided. Accordingly, lumen enlargement is used as a practical endpoint of OA (Figure 5). In some situations, IVUS marking is important for identifying the target debulking area, as Glide Assist mode may allow the device to cross the lesion without sufficient plaque modification (Figure 6). The use of IVUS does not require blood clearance during imaging. Therefore, IVUS is particularly valuable in ostial lesions, in which guidewire position may be influenced by guide catheter engagement. In addition, strict coaxial alignment of the guide catheter is not mandatory for OA treatment (Figure 7).

#### 3.3.2. OCT Guidance

**Figure 8:**
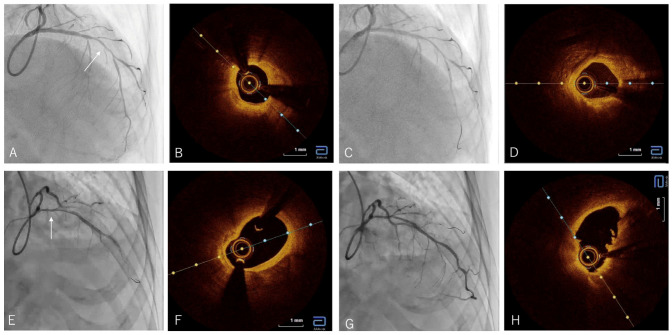
Guidewire position and OA from both branches. (**A**) Angiogram. (**B**) OCT demonstrating calcium distributed from the 1 to 6 o’clock positions. The guidewire from the LAD–LAD branch was positioned at the 3 o’clock position, whereas the guidewire from the diagonal–LAD branch was positioned at the 11 o’clock position. OA was performed using the LAD–LAD. (**C**) Angiogram after OA at 80,000 rpm. (**D**) OCT after OA at 80,000 rpm demonstrating effective calcium modification. (**E**) Angiogram (**F**) OCT demonstrating calcium distributed from the 4 to 9 o’clock positions. The guidewire from the LAD–LAD branch was positioned at the 12 o’clock position, whereas the guidewire from the diagonal–LAD branch was positioned at the 7 o’clock position. OA was performed using the diagonal–LAD. (**G**) Angiogram after OA at 80,000 rpm. (**H**) OCT demonstrating effective calcium modification. White arrows indicate the lesion.

If intravascular imaging is feasible, the extent of calcification amenable to modification can be estimated. When performing OA in cases in which a bifurcation is located distal to the culprit lesion, it is important to determine which distal guidewire position is safer and more effective. By withdrawing the imaging device from both branches, optical coherence tomography (OCT) can be used to assess which branch—or whether both branches—should undergo OA. This approach enables safe and effective use of OA while reducing the risk of vessel injury (Figure 8).

**Figure 9:**
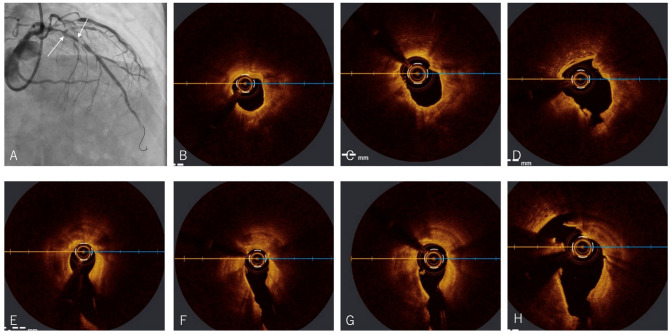
OCT-guided OA. (**A**) Angiogram (**B**) OCT identified severe calcification in the proximal portion. Thickness of calcium at 6 o’clock was 980 μm. (**C**) 80,000 rpm OA modified both calcium (620 μm) and fibrous plaque (10–12 o’clock). (**D**) 3.0 mm scoring balloon made fracture at 6 o’clock. (**E**) OCT identified severe calcification in the distal portion. Thickness of Calcium at 12 o’clock was 1170 μm. (**F**) 80,000 rpm OA modified calcium (1020 μm) (**G**) 120,000 rpm OA modified calcium (650 μm) (**H**) 3.0 mm scoring balloon made fractures at 12 and 3 o’clock. White arrows indicate the lesion.

It is generally important to mark the target ablation area using angiographic coregistration. In superficial calcified sheets, calcification thickness is an important determinant of subsequent treatment strategy. The potential extent of modification can be roughly estimated based on the relative positions of the imaging catheter and guidewire on OCT. Therefore, detailed planning with angiographic coregistration is necessary to achieve more effective and safer modification (Figure 9).

Typically, intravascular imaging is performed after OA at 80,000 rpm and before escalation to 120,000 rpm to assess the adequacy of plaque modification [20,21]. When calcification is reduced to a thickness of approximately 500–700 μm using OA, fracture of the calcified plaque can be facilitated with a cutting balloon, scoring balloon, or high-pressure balloon. For DES implantation, achieving the desired reduction in calcified tissue thickness may be considered the endpoint of OA. The potential effectiveness of OA can be partially predicted based on the spatial relationship between the guidewire and the imaging catheter. Caution is required when evaluating calcified lesions in which the guidewire is positioned close to fibrous or soft plaque on OCT, as plaque modification may extend beyond the intended area by passing Glide Assist mode.

### 3.4. Potential Use of OA

#### 3.4.1. Tortuous Lesions

Coronary artery tortuosity has been associated with increased procedural complexity and lower immediate success rates because of difficulty in guidewire manipulation and delivery of angioplasty equipment to the target lesion [22,23]. In typical tortuous lesions, calcification is often located along the lesser curvature of the vessel. With RA, there is an increased risk of dissection and perforation because the burr tends to track along the greater curvature of the vessel in tortuous segments. The presence of acute angulation at the lesion site has been reported to be associated with procedural failure of RA, as advancing the burr across such lesions increases the risk of burr entrapment or vessel perforation [24,25,26,27]. Therefore, a limited or halfway RA approach is often recommended for severely tortuous lesions. In contrast, OA serves both antegrade and retrograde action. Particularly in tortuous lesions, retrograde OA moves the lesser curvature of the vessel, then adequate modification of calcium can be accomplished.

**Figure 10:**
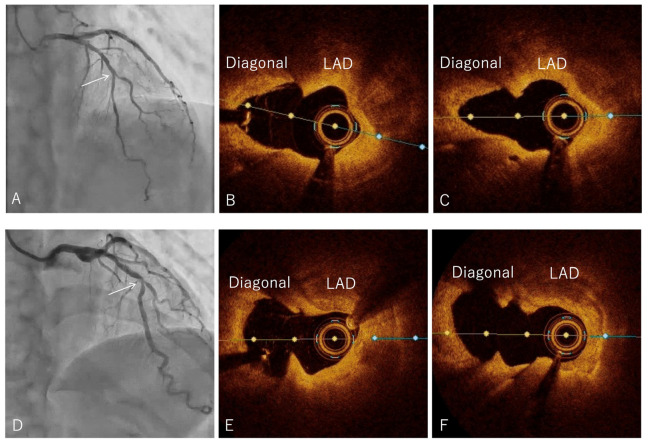
Comparison between RA and OA in a tortuous vessel. (**A**) Angiography (**B**,**C**) OCT before and after 1.75 mm RA. (**D**) Angiography (**E**,**F**) OCT before and after 80,000 rpm OA. White arrows indicate the lesion. LAD: Left anterior descending artery, Diagonal: Diagonal branch.

**Figure 11:**
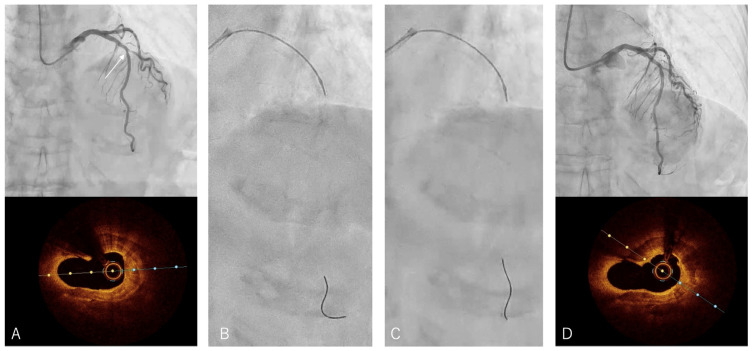
Position of the OA crown. (**A**) Angiogram and OCT. (**B**) OCT demonstrating calcification located opposite the diagonal branch. (**C**) After slight release of the guidewire, the OA crown shifted toward the calcified plaque on angiography. (**D**) Retrograde OA was performed for this lesion. Angiography and OCT after OA at 80,000 rpm demonstrated effective modification of the calcified lesion. The white arrow indicates the lesion.

In such cases, the OA catheter should be advanced to the distal segment using Glide Assist mode, followed by retrograde OA (Figure 10). In some instances, partial withdrawal and repositioning of the ViperWire before OA may help shift the guidewire toward the lesser curvature of the tortuous coronary vessel (Figure 11).

#### 3.4.2. Long Lesions

**Figure 12:**
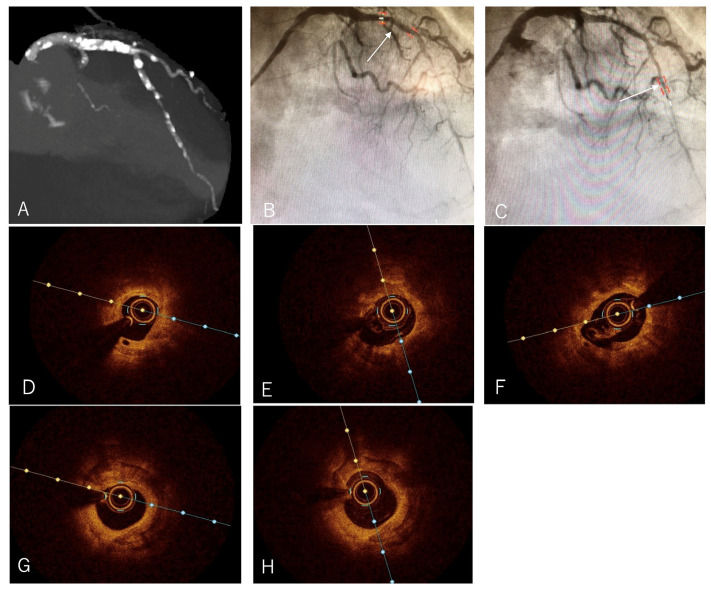
OA for a long lesion. (**A**) Computed tomography angiography (CTA). (**B**) OCT guidance identifying the proximal OA target site. (**C**) OCT guidance identifying the distal OA target site. (**D**) Proximal OCT before OA. (**E**) Proximal OCT after OA at 80,000 rpm. (**F**) Calcium thickness was still more than 800 μm at 10–11 o’clock so 120,000 rpm OA was applied to the proximal lesion. Proximal OCT after OA at 120,000 rpm. (**G**) Distal OCT before OA. (**H**) Distal OCT after OA at 80,000 rpm. Calcium thickness was less than 400 μm, so 120,000 rpm OA was not employed. White arrows indicate the lesion. Red lines indicate the modification area of OA.

Atherectomy with OA generally requires a longer treatment time than RA, and OA is not typically used to modify the entire length of a long lesion. Although RA may be considered in cases of diffuse calcification involving long segments, OA may be preferable when there is concern regarding unfavorable guidewire bias or when modification of adjacent soft plaque poses additional risk during RA. In such cases, marking techniques using IVUS or OCT are important. The use of intravascular imaging to delineate and mark the target modification area is essential to reduce complications and achieve adequate modification with OA (Figure 12).

#### 3.4.3. Ostial Lesions

Evaluation of the ostial right coronary artery (RCA) using OCT is often challenging. Confirmation of a safe guide catheter (GC) position using IVUS, followed by OA under these conditions, allows adequate plaque modification. OA appears to be a feasible and safe treatment option for calcified coronary ostial lesions. Treatment of ostial lesions remains challenging, and procedural success and clinical outcomes are inferior compared with non-ostial lesions, mainly because of the high prevalence of elastic fiber content, eccentric calcium burden, and vessel angulation. Recent reports of RA have demonstrated high rates of major adverse cardiac events (MACE). With RA, proper GC engagement is required and antegrade RA is generally performed. Although recent reports have recommended ensuring coaxial alignment of the GC, strong GC backup support is not mandatory when using an OA strategy. IVUS examination can confirm appropriate GC positioning for OA. With IVUS guidance, the risk of aorto-ostial injury or perforation may be substantially reduced. After the OA crown crosses the ostial lesion in Glide Assist mode, either a floating or engaged GC position can be selected based on IVUS findings, and the device should then be activated at low speed in a retrograde manner. Following low-speed OA, IVUS reassessment should be performed with evaluation of different GC positions. Adequate calcium modification can subsequently be achieved.

**Figure 13:**
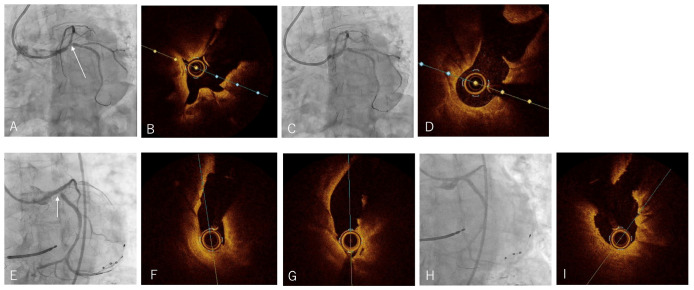
Calcified ostial LCx lesion. (**A**,**B**) Angiography and OCT before OA demonstrating a noneruptive calcified nodule in the ostial LCx. (**C**,**D**) Angiography and OCT after retrograde OA at 80,000 rpm. (**E**–**G**) Angiography and OCT after additional retrograde OA at 80,000 rpm. (**H**,**I**) Angiography and OCT after antegrade OA at 80,000 rpm. White arrows indicate the lesion.

Depending on guidewire location, if the LCx ostial lesion demonstrates sheet-like calcification, it is usually situated on the side opposite the LAD, making retrograde OA advantageous. However, the calcium is located on the carina side in some cases. In such cases, antegrade OA is effective to modify calcium (Figure 13). The priority of OA is to select antegrade or retrograde fashion depending on the calcium location.

#### 3.4.4. Bifurcation Lesions

**Figure 14:**
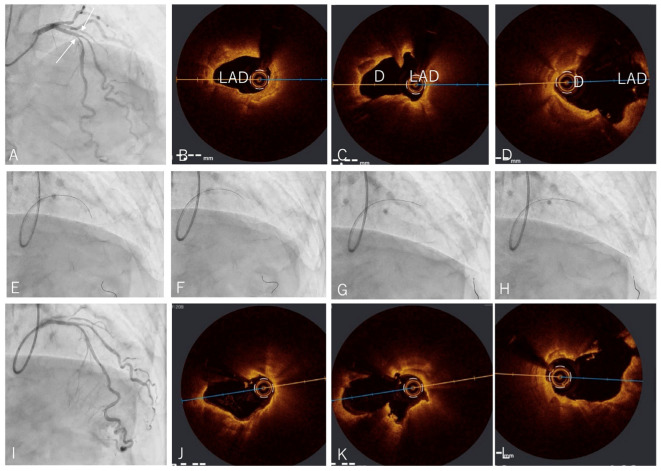
OA for a calcified bifurcation lesion. (**A**–**D**) Angiography and OCT demonstrating the proximal segment, LAD bifurcation, and diagonal bifurcation. (**E**,**F**) Retrograde OA at 80,000 rpm from LAD to LAD. (**G**,**H**) Retrograde OA at 80,000 rpm from the diagonal branch to the LAD. (**I**–**L**) Angiography and OCT of the proximal segment, LAD bifurcation, and diagonal bifurcation after retrograde OA at 80,000 rpm. White arrows indicate the lesion.

Compared with nonbifurcation lesions, bifurcation lesions are associated with higher rates of MACE. Calcified bifurcation lesions are reported to have an even poorer prognosis after treatment [28]. One potential concern in bifurcation lesions is the risk of side branch (SB) compromise following DES implantation. In addition to main branch (MB) plaque burden, calcification on the side opposite the SB and stenosis at the SB ostium are thought to influence the risk of SB compromise after MB stent placement [29,30,31]. Since bifurcation lesions introduce procedural challenges related to maintaining SB patency, adequate lesion preparation—including modification of calcified plaque with a plaque-modifying device—is essential [32,33,34]. Because RA is largely influenced by guidewire bias, the modification site can be predicted to some extent using procedural intravascular imaging. Imaging devices can be used to determine whether OA should be performed in one or both branches. This strategy enables safe and effective OA while reducing procedural risk (Figure 14).

#### 3.4.5. LMCA Lesions

**Figure 15:**
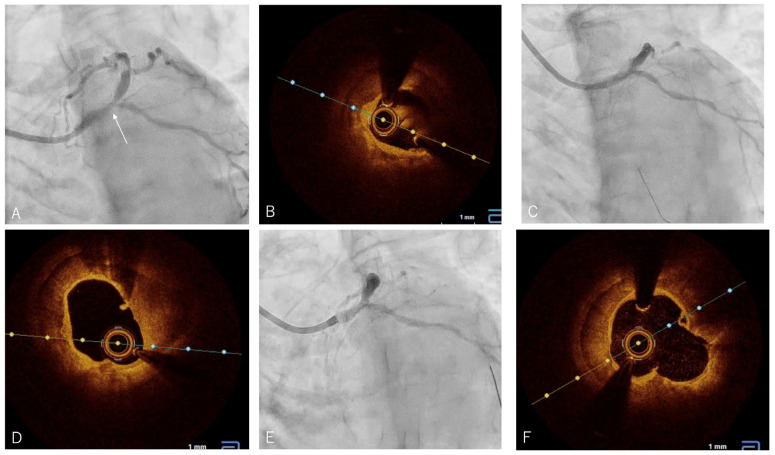
Calcified LMCA lesion. (**A**) Angiogram. (**B**) OCT demonstrating that the guidewire from the LAD was positioned at the 5 o’clock position and that from the LCx at the 10 o’clock position, the lumen area measured 2.80 mm^2^. (**C**,**D**) After OA at 120,000 rpm from the LAD to the LMCA, the lumen area increased to 3.80 mm^2^, with plaque modification primarily between the 3 and 6 o’clock positions. (**E**,**F**) After OA at 120,000 rpm from the LCx to the LMCA, the lumen area increased to 7.12 mm^2^. The white arrow indicates the lesion.

Because the LMCA is the largest vessel in the coronary tree, plaque volume and calcification are generally greater than in other coronary segments and often extend to the distal bifurcation. Left main coronary artery disease represents a complex pathological condition associated with increased morbidity and mortality because of the large myocardial territory at risk for ischemia [35]. In addition, calcified plaque often extends contiguously into the LAD and/or the LCx. Because guidewire positioning from the LAD and LCx frequently differs, greater lumen gain through calcium modification may be achieved by performing OA from both the LAD–LMCA and LCx–LMCA directions (Figure 15).

### 3.5. Eruptive Calcified Nodules

**Figure 16:**
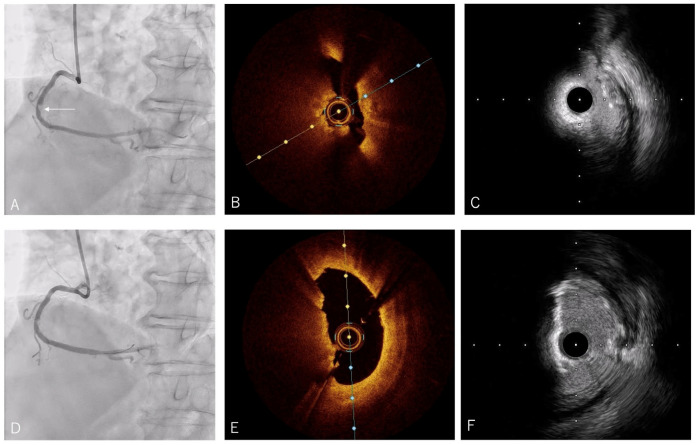
OA for an eruptive calcified nodule. (**A**) Angiogram. (**B**,**C**) OCT and IVUS demonstrating an eruptive calcified nodule. Guidewire position is located near eruptive calcified nodule. (**D**) Angiogram after OA at 120,000 rpm. (**E**,**F**) OCT and IVUS demonstrating modification of the eruptive calcified nodule after OA at 120,000 rpm. The white arrow indicates the lesion.

**Figure 17:**
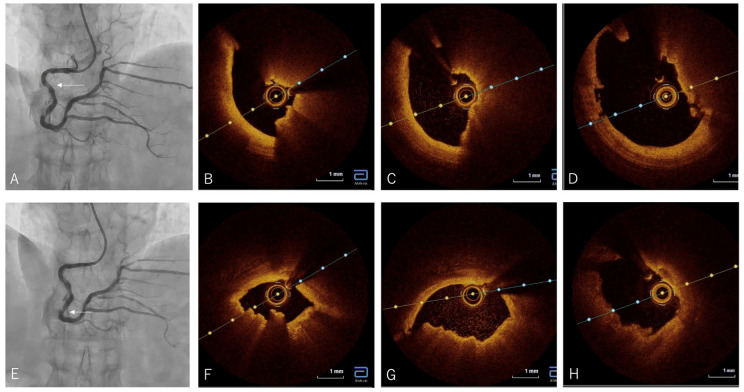
OA for an eruptive calcified nodule (**A**) Angiogram (**B**) OCT showed that the guidewire position (3 o’clock) at the proximal site was located near eruptive calcified nodule. (**C**) After OA at 80,000 rpm, OCT demonstrating modification of eruptive calcified nodule. (**D**) Final 4.0 mm DCB was applied. (**E**) Angiogram (**F**) OCT showed that the guidewire position (12 o’clock) at the distal site was located near vessel wall and far from eruptive calcified nodule. (**G**) Because the guidewire position was not sufficient for OA, 3.5 mm IVL was applied to this lesion. (**H**) Final 4.0 mm DCB was applied. White arrows indicate the lesion.

Management of eruptive calcified nodules (CNs) remains challenging for interventional cardiologists because these lesions represent a manifestation of severe coronary calcification and are associated with adverse clinical outcomes. As eruptive CNs may occur in large-caliber and/or highly tortuous vessel segments, the eccentrically mounted OA crown can provide bidirectional sanding or retrograde-only modification through centrifugal force, potentially achieving safer lumen enlargement compared with RA [36,37] (Figure 16). Previous study showed OA, RA, and IVL had a similar effect on calcified nodules; however, they did not demonstrate the relation between guidewire position and the effect of devices. [38,39]. If the guidewire position is not sufficient for OA, OA should not be performed in these lesions because of high risk of vessel injury (Figure 17).

### 3.6. Complications

#### 3.6.1. Complication Associated with OA Catheter Systems

##### OA ViperWire Dislodgement

**Figure 18:**
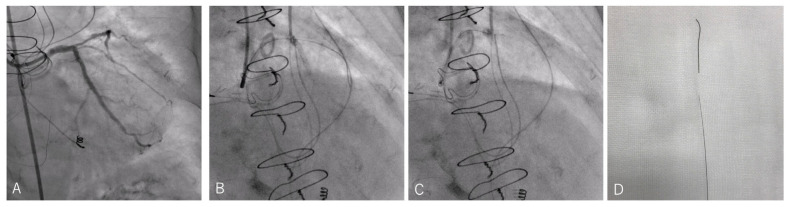
ViperWire dislodgement during Glide Assist mode. (**A**) Angiogram. (**B**) ViperWire insertion. (**C**) During Glide Assist mode of OA, the ViperWire was retracted and subsequently dislodged. (**D**) The ViperWire was easily removed.

During Glide Assist mode, the brake is released, and the ViperWire may advance or retract unintentionally. If Glide Assist is activated under these circumstances, ViperWire dislodgement may occur. If the ViperWire retracts proximally, further displacement may be prevented by immediately pressing the Glide Assist button to halt rotation. When OA ViperWire dislodgement occurs, removal of the system followed by recrossing with another guidewire represents one bailout strategy (Figure 18).

##### OA Crown Dislodgement and Entrapment

**Figure 19:**
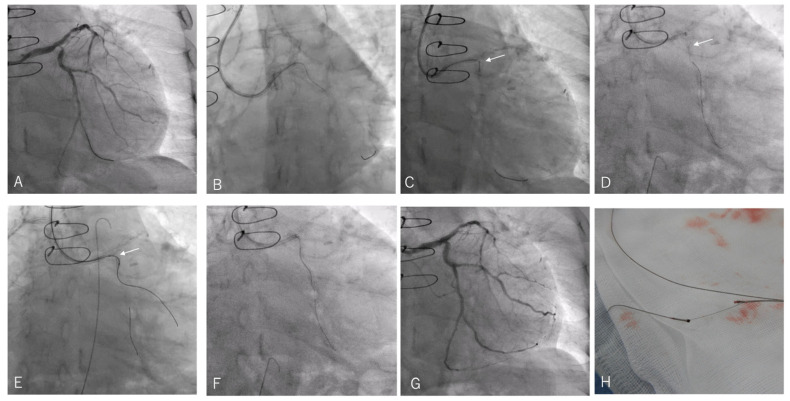
OA crown dislodgement. (**A**) Angiogram. (**B**) The OA catheter was advanced with a guide extension catheter. (**C**,**D**) During retrograde OA, contact between the guide extension catheter and the OA crown resulted in crown dislodgement. The crown could not initially be retrieved. (**E**,**F**) Another regular guidewire was advanced, and traditional balloon angioplasty was performed. (**G**) Angiogram after retrieval of the crown. (**H**) The retrieved OA crown. The white arrow indicates the dislodged crown.

Dislodgement of the OA catheter may occur in the following situations: (1) when the catheter is forcibly advanced in an antegrade manner, resulting in entrapment. This mechanism is similar to RA-related entrapment. In this situation, forceful retraction may lead to device dislodgement. (2) When the catheter is forcibly retracted in a retrograde manner, dislodgement may occur at or near the site of entrapment. (3) When the GC or guide extension catheter is not coaxially aligned with the OA catheter during retrograde retrieval, dislodgement may occur because of misalignment between the systems (Figure 19).

### 3.7. Vascular Complications Associated with OA

#### Slow Flow and No-Reflow

Because the eccentrically mounted crown sands plaque from the outer surface while maintaining blood flow, coronary perfusion is generally preserved during OA. However, in severe lesions, coronary flow may be transiently reduced. In such situations, there remains a risk of slow flow even with OA. In addition, prolonged OA runs may increase the risk of slow flow; therefore, each OA run should be limited to approximately 30 s. When prolonged lesion crossing is anticipated, staged plaque modification—consisting of partial modification, repositioning to the original platform using the engage-and-release technique, and subsequent repeat ablation—may reduce the risk of slow flow and device entrapment.

### 3.8. Bradycardia and Hypotension

Coronary flow is generally maintained during OA, and bradycardia and hypotension occur less frequently than with RA. However, in RCA lesions with preexisting bradycardia or in high-risk cases, prophylactic administration of atropine or aminophylline may be considered.

### 3.9. Differential Sanding

**Figure 20:**
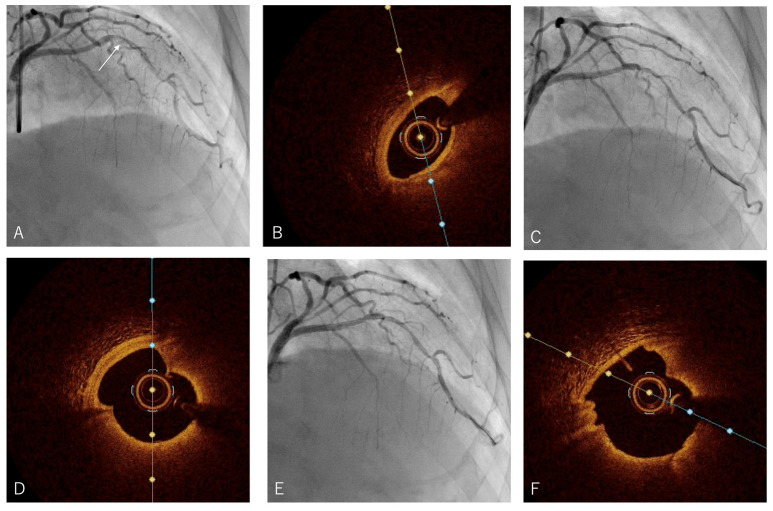
Limited differential sanding effect with OA. (**A**,**B**) Angiography and OCT before OA. OCT demonstrated a three-layer structure between the 7 and 12 o’clock positions. (**C**,**D**) Angiography and OCT after OA at 80,000 rpm. OCT demonstrated persistence of a three-layer structure between the 9 and 12 o’clock positions, with effective modification of eccentric calcification. (**E**,**F**) Angiography and OCT after OA at 120,000 rpm. OCT demonstrated disappearance of the three-layer structure between the 9 and 12 o’clock positions, reflecting greater modification of eccentric calcification. The white arrow indicates the lesion.

When a three-layer structure is observed on OCT, differential sanding may be preserved, similar to the differential cutting mechanism described in RA. However, this effect is limited and not absolute. Therefore, if the guidewire is positioned close to a noncalcified lesion, OA is not recommended in such cases (Figure 20).

### 3.10. Dissection, Perforation, and Pseudoaneurysm

In soft plaques, the centrifugal force generated by OA may readily induce dissection; therefore, caution is required to avoid excessive modification, even when the plaque is distant from the guidewire. In fibrous plaque, OA may also result in unpredictable tissue modification; thus, OA should be avoided when the guidewire is positioned close to fibrous tissue. Because plaque reduction is more easily achieved in eccentrically distributed lesions than in densely calcified lesions, careful procedural planning is necessary.

## 4. Conclusions

Severe coronary calcification represents a major limitation in PCI. OA and RA primarily rely on tissue modification and are used to modify coronary calcified lesion. Where RA burrs spin concentrically on the guidewire and perform front-cutting, the OA crown uses centrifugal force and bi-directional sanding. These differences may lead to different calcium modification and vessel injury. Prevention of complications in OA requires understanding its mechanism.

## Figures and Tables

**Table 1:** Comparison of coronary artery modification devices.

Parameter	Orbital Atherectomy	Rotational Atherectomy
Mechanism of Action	Eccentrically mounted diamond-crown uses centrifugal force to orbit	Rotational diamond-tipped burr spins concentrically on the wire
Crown/Burr size	1.25 mm (Crown)	1.25–2.50 mm (Burr)
Guidewire bias	Yes	Yes
Non-guidewire bias	Yes	No
Continuous blood flow	Yes	No
Ability of forward and backward	Bi-directional sanding	Front cutting

## Data Availability

No new data were created or analyzed in this study.
